# HBK-14 and HBK-15 with antidepressant-like and/or memory-enhancing properties increase serotonin levels in the hippocampus after chronic treatment in mice

**DOI:** 10.1007/s11011-016-9932-9

**Published:** 2016-11-25

**Authors:** Karolina Pytka, Katarzyna Gawlik, Dorota Pawlica-Gosiewska, Jadwiga Witalis, Anna Waszkielewicz

**Affiliations:** 1 Department of Pharmacodynamics, Faculty of Pharmacy, Jagiellonian University Medical College, Medyczna 9, 30-688 Krakow, Poland; 2 Department of Diagnostics, Chair of Clinical Biochemistry, Jagiellonian University Medical College, Krakow, Poland; 3 Department of Bioorganic Chemistry, Chair of Organic Chemistry, Faculty of Pharmacy, Jagiellonian University Medical College, Medyczna 9, 30-688 Krakow, Poland

**Keywords:** 2-methoxyphenylpiperazine derivative, 5-HT_1A_ receptor antagonist, 5-HT_7_ receptor antagonist, Forced swim test, Step-through passive-avoidance test, Mice

## Abstract

5-HT_1A_ and 5-HT_7_ receptor ligands might have antidepressant-like properties and improve cognitive function. We previously reported significant antidepressant- and anxiolytic-like effects of two dual 5-HT_1A_ and 5-HT_7_ receptor antagonists in various behavioral tests in rodents. As a continuation of our previous experiments, in this study we aimed to investigate whether chronic administration of 1-[(2,6-dimethylphenoxy)ethoxyethyl]-4-(2-methoxyphenyl)piperazine hydrochloride (HBK-14) and 1-[(2-chloro-6-methylphenoxy)ethoxyethyl]-4-(2-methoxyphenyl)piperazine hydrochloride (HBK-15) caused antidepressant-like effects and elevated serotonin levels in the murine hippocampus. We also evaluated cholinolytic properties and the influence of acute administration of both compounds on cognitive function in mice. To assess antidepressant-like properties and the influence on learning and memory we used forced swim test and step-through passive avoidance task in mice, respectively. Both compounds showed antidepressant-like properties and significantly elevated serotonin levels in the hippocampus after chronic treatment (HBK-14 – 2.5 mg/kg; HBK-15 – 0.625 and 1.25 mg/kg). HBK-15 administered chronically antidepressant-like activity at lower dose (0.625 mg/kg) than the dose active after acute treatment (1.25 mg/kg). None of the compounds affected locomotor activity of mice. HBK-15 possessed very weak cholinolytic properties, whereas HBK-14 did not show any effect on muscarinic receptors. Only HBK-15 (0.625 mg/kg) presented memory-enhancing properties and ameliorated cognitive impairments caused by scopolamine (1 mg/kg). Our results indicate that 5-HT_1A_ and 5-HT_7_ antagonists might have potential in the treatment of depression and possess positive influence on cognitive function.

## Introduction

Major depression affects millions of people worldwide and contributes to their disability. Besides the well-defined depressive symptoms, patients often report cognitive disturbances, which significantly deteriorate their functioning. Therefore, the scientists still search for the new compounds with increased efficacy and positive influence on cognition.

Current antidepressants worsen (e.g. tricyclic antidepressants) or have no influence (e.g. selective serotonin reuptake inhibitors) on cognitive function (for review see: Biringer et al. 2009). Some Authors suggested that reboxetine, bupropion, duloxetine or venlafaxine might have more beneficial effect on cognitive function than other antidepressants. However, recent meta-analysis of clinical trials showed that only vortioxetine significantly improved cognition in depressed patients (McIntyre et al. 2016). Positive influence on cognitive function was most likely due to the drug’s broad receptor profile. Studies on animals showed that both 5-HT_1A_ and 5-HT_7_ receptor ligands might enhance cognitive function in rodents (reviewed in Glikmann-Johnston et al. 2015 and Meneses 2014). Interestingly, 5-HT_1A_ antagonism facilitated memory retention possibly via 5-HT_7_ activation, and 5-HT_7_ receptor could improve emotional memory upon reduced 5-HT_1A_ receptor transmission (Stiedl et al. 2015).

We previously reported significant antidepressant- and anxiolytic-like effects of two dual 5-HT_1A_ and 5-HT_7_ receptor antagonists in various behavioral tests in rodents (Waszkielewicz et al. 2015; Pytka et al. 2015a). As a continuation of our previous experiments, in this study we aimed to investigate whether chronic administration of 1-[(2,6-dimethylphenoxy)ethoxyethyl]-4-(2-methoxyphenyl)piperazine hydrochloride (HBK-14) and 1-[(2-chloro-6-methylphenoxy)ethoxyethyl]-4-(2-methoxyphenyl)piperazine hydrochloride (HBK-15) caused antidepressant-like effects and elevated serotonin levels in the murine hippocampus. We also evaluated cholinolytic properties and the influence of acute administration of both compounds on cognitive function in mice.

## Materials and methods

### Animals

Adult male mice (stock’s name: CD-1, 18–21 g), purchased from Animal House at the Faculty of Pharmacy, Jagiellonian University Medical College, Krakow, Poland or and male guinea-pigs (Outbred CV, 300-400 g), purchased from Laboratory Animals Husbandry Maria Staniszewska, Słaboszów, Poland were used in the experiments. The groups of 15 mice or 2 guinea-pigs were kept to a plastic cage (60 cm × 38 cm × 20 cm) at a room temperature (22 ± 2 °C), on 12 h light/dark cycles (the lights turned on at 7:00 a.m., and off at 19:00 p.m.). Animals had free access to standard laboratory food and tap water. Behavioral experiments were performed between 9 a.m. and 2 p.m. and each experimental group consisted of 8–10 randomly selected animals. In acute experiments mice were used only once in each test. After the experiment mice were killed by cervical dislocation. Guinea-pigs were anaesthetized (37 mg/kg sodium pentobarbital) and killed by cervical dislocation. All experimental procedures were carried out in accordance with EU Directive 2010/63/EU and approved by the I Local Ethics Committee for Experiments on Animals of the Jagiellonian University in Krakow, Poland (approval numbers: 52/2014 and 102/2015)*.*

### Drugs

**Fig. 1:**
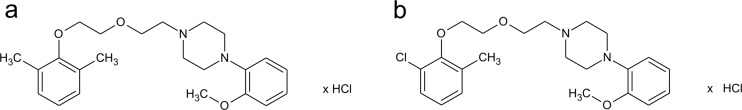
Chemical structures of the studied dual 5-HT_1A_ and 5-HT_7_ receptor antagonists. **a** 1-[(2,6-dimethylphenoxy)ethoxyethyl]-4-(2-methoxyphenyl)piperazine hydrochloride (HBK-14); **b** 1-[(2-chloro-6-methylphenoxy)ethoxyethyl]-4-(2-methoxyphenyl)piperazine hydrochloride (HBK-15)

The studied compounds (Fig. 1): 1-[(2,6-dimethylphenoxy)ethoxyethyl]-4-(2-methoxyphenyl)piperazine hydrochloride (HBK-14) and 1-[(2-chloro-6-methylphenoxy)ethoxyethyl]-4-(2-methoxyphenyl)piperazine hydrochloride (HBK-15) were synthesized in the Department of Bioorganic Chemistry, Chair of Organic Chemistry, Pharmaceutical Faculty, Jagiellonian University (Waszkielewicz et al. 2015). HBK-14, HBK-15, and scopolamine (Sigma, Germany) were dissolved in saline, and administered intraperitoneally (i.p.) at a volume of 10 ml/kg. Carbachol (Sigma, Germany) was dissolved in distilled water and used in functional experiments.

### Experimental protocol for chronic experiments

**Fig. 2:**
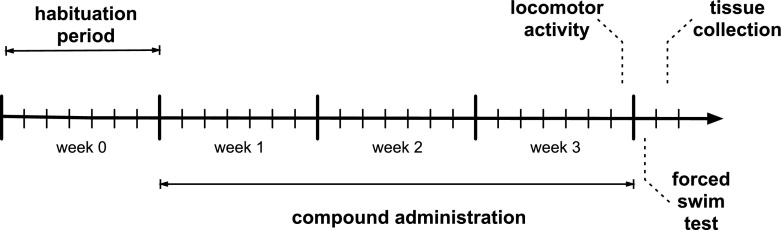
A schematic diagram of chronic administration of studied compounds and behavioral testing. HBK-14 (1.25 and 2.5 mg/kg), HBK-15 (0.3, 0.625 and 1.25 mg/kg), fluoxetine (10 mg/kg) or saline were administered intraperitoneally to mice for 21 consecutive days. Control groups received 0.9% NaCl (saline)

After habituation period, mice were injected (i.p.) with HBK-14 (1.25 or 2.5 mg/kg), HBK-15 (0.3, 0.625 or 1.25 mg/kg), fluoxetine (10 mg/kg – positive control) or saline (control groups) for 21 consecutive days (Fig. 2). After that time the animals were subjected to behavioral tests (i.e. forced swim test and spontaneous locomotor activity), which were then followed by tissue collection and biochemical analysis. The doses of compounds used in this study were based on our previous experiments (Pytka et al. 2015b; Pytka et al. 2015a).

#### Forced swim test

Forced swim test was performed according to the method previously described (Borsini 1995; Pytka et al. 2015c) with some modifications (Tanaka and Telegdy 2008; Pytka et al. 2015c; Pytka et al. 2016a). Mice were placed individually in glass cylinders (height 25 cm, diameter 10 cm) containing 10 cm^3^ of water (23–25 °C), and left there for 6 min. The total time of immobility, swimming and climbing was recorded during the final 4 min of the test. The animal was regarded as: 1) immobile when it was floating passively in the water or making only small movements to keep its head above the water surface; 2) climbing when it was making vertical movements against walls; 3) swimming when it was making horizontal movements across the water surface. The experiments were recorded and scored using aLab.io software by a trained observer blind to the treatments.

#### Spontaneous locomotor activity

The locomotor activity was performed as previously described (Pytka et al. 2015b). Briefly, locomotor activity was recorded for each animal individually using activity cages, which were supplied with I.R. horizontal beam emitters (Activity Cage 7441, Ugo Basile, Italy) connected to a counter for the recording of light-beam interruptions. Each mouse was placed in a cage (40 cm × 40 cm × 31 cm), made of clear Perspex, for a 30 min habituation period. After that time the number of crossings of photobeams was measured from the 2nd to the 6th min (i.e., the time equal to the observation period in the forced swim test). The cages were disinfected with 70% ethanol after each mouse.

#### The evaluation of serotonin level in the hippocampus

Removed hippocampi were rinsed with phosphate-buffered saline (PBS) to remove excess blood. The tissue was homogenized in PBS buffer enriched with 1% stabilizer, included in the kit, and stored in -80 °C. After two freeze-thaw cycles that were performed to break the cell membranes, the homogenates were centrifuged for 10 min at 4000 x g. Removed supernate were aliquoted and stored at -80 °C until analysis.

Aliquots of each sample were taken and serotonin levels were determined using an ELISA kit from Labor Diagnostyka Nord GmbH &Co. KG (LDN, Nordhorn, Germany), according to the manufacturer’s instructions. The absorbance was read at 450 nm and the corresponding concentration was determined from the standard curve.

The serotonin level in murine hippocampi was additionally evaluated after single injection.

### The influence on the guinea-pig ileum contraction induced by carbachol

The experiment was performed according to the method described by Mogilski and colleagues (2015). A segment (15 cm) of male guinea-pig ileum was excised from the small intestine and immersed into a Krebs solution (NaCl 120 mM, KCl 5.6 mM, MgCl_2_ 2.2 mM, CaCl_2_ 2.4 mM, NaHCO_3_ 19 mM, glucose 10 mM). The part of the ileum (5 cm) that was the closest to the ileo-cecal junction was removed. After 2 cm-long fragments of the ileum were cut, each of them was placed in 20 ml chamber of tissue organ bath system (Tissue Organ Bath System — 750 TOBS, DMT, Denmark) filled with the Krebs solution at 37 °C, pH 7.4, with constant oxygenation (O_2_/CO_2_, 19:1). The segments were stretched by means of closing clips between the metal rod and the force–displacement transducer. The preparations were allowed to stabilize in organ baths for 60 min under a resting tension of 0.5 g and were washed every 15 min with fresh Krebs solution. After the equilibration period a cumulative concentration–response curve was constructed for muscarinic receptor agonist: carbachol (3 nM – 3 μM). Then the tissues were incubated with one of the concentrations of tested compounds for 15 min and the next cumulative concentration curve to the agonist was constructed. Only one concentration of the potential antagonist was tested in each piece of the tissue. The experiment was repeated four to eight times.

### Step-through passive avoidance task in mice after acute administration

Step-through passive avoidance task was performed according to the method previously described (Lee et al. 2016; Pytka et al. 2016a). The apparatus for step-through passive avoidance task consisted of two compartments, separated by an automated sliding door (LE872, Bioseb, France). For acquisition session, mice were placed individually in an illuminated white compartment (20 cm × 21 cm × 20 cm, 1000 lx) with the closed door to a smaller dark compartment (7.3 cm × 7.5 cm × 14 cm, 10 lx) equipped with an electric grid floor (stainless steel rods through which an electric footshock is delivered). After 30 s the door to a smaller compartment were opened. Immediately after the mouse entered the smaller dark compartment, the door closed and the rodent was punished by an inescapable electric foot shock (0.8 mA for 2 s). The mice, which did not enter the dark compartment within 50 s were excluded from the study. On the following day (24 h later), the pretrained animals were placed again into the illuminated compartment and observed up to 300 s (retention session). The experimental procedure was similar to acquisition session, but this time mice did not receive the electric shock after the entrance to the smaller dark compartment. Mice, which avoided the dark compartment for 300 s were considered to remember the task.

#### Evaluation of cognitive-enhancing properties in naïve mice

HBK-14 (1.25 or 2.5 mg/kg), HBK-15 (0.3, 0.625 or 1.25 mg/kg) or fluoxetine (5, 10, 20 mg/kg) were administered (i.p.) 30 min before acquisition trial. Control groups were injected (i.p.) with saline. The doses of compounds used in this study were based on our previous experiments (Pytka et al. 2015b; Pytka et al. 2015a).

#### The effect on scopolamine-induced cognitive dysfunction

HBK-14 (1.25 or 2.5 mg/kg), HBK-15 (0.3, 0.625 or 1.25 mg/kg) or fluoxetine (5, 10, 20 mg/kg) and scopolamine (1 mg/kg) were administered (i.p.) 30 min before the acquisition trial. Control groups were injected (i.p.) with saline. The doses of the studied compounds and scopolamine were based on our previous experiments (Pytka et al. 2015b; Pytka et al. 2015a; Pytka et al. 2016a).

### Data analysis

Results are presented as means ± S.E.M. In the forced swim test, spontaneous locomotor activity, and biochemical studies comparisons between experimental and control groups were performed by one-way ANOVA, followed by Newman-Keuls post hoc, when three or more groups were compared or Student’s t-test if two groups were compared. In the step-through passive avoidance task one-way ANOVA followed by Turkey’s test post hoc was used. A value of *p* < 0.05 was considered to be significant.

In functional experiments pK_B_ value was estimated using the following equation (Arunlakshana and Schild 1959):

$$ p{K}_B=-{ \log}_{10}\frac{B}{DR-1} $$

Where B was the molar antagonist concentration and DR was the ratio between the EC_50_ of the agonist in the presence and absence of the antagonist. pK_B_ value was equivalent to the pA_2_ value and was calculated if only one concentration of tested compound was effective.

## Results

### Antidepressant-like activity of studied compounds and fluoxetine in the forced swim test in mice after chronic treatment

**Fig. 3:**
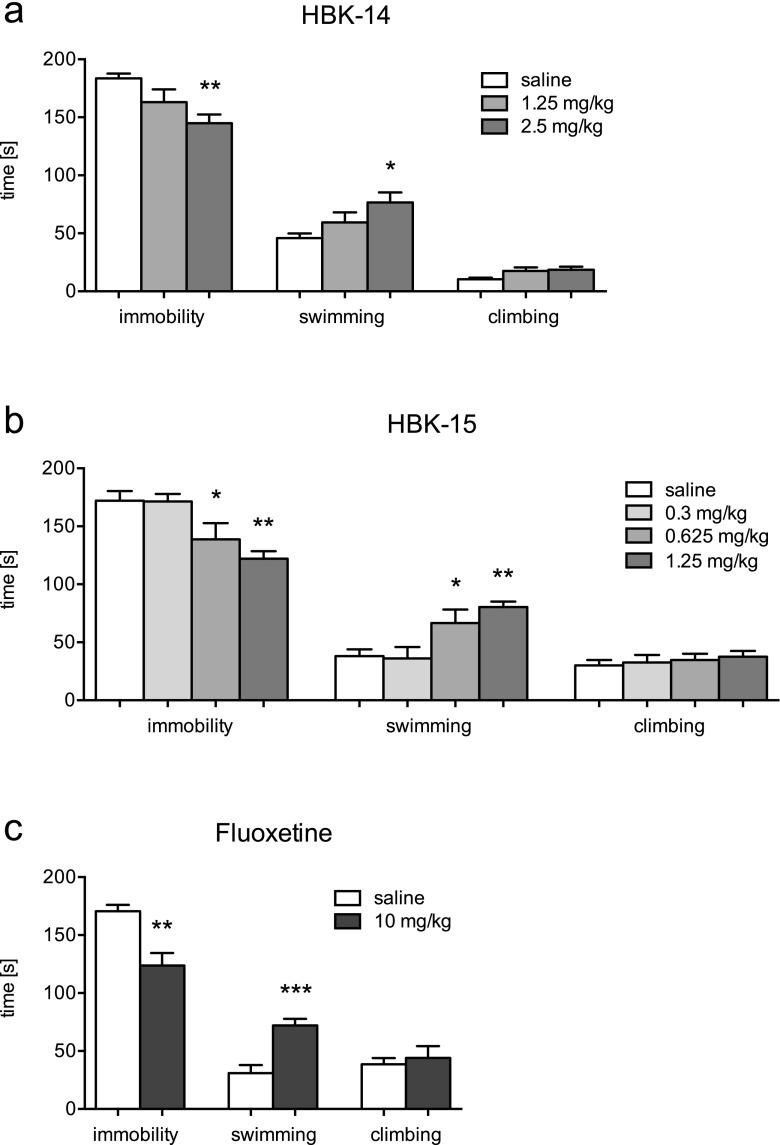
Antidepressant-like effect of HBK-14, HBK-15 and fluoxetine after chronic treatment in mice. HBK-14, HBK-15, fluoxetine or saline were administered intraperitoneally to mice for 21 consecutive days. 24 h after the last injection the forced swim test was performed. Control groups received 0.9% NaCl (saline). Statistical analysis: one-way ANOVA (Newman-Keuls post hoc) when three or more groups were compared or Student t-test if two groups were compared, *p < 0.05, **p < 0.01, ***p < 0.001 vs respective saline-treated group; *n* = 8 mice per group

HBK-14 (2.5 mg/kg) injected for 21 days significantly reduced immobility of mice by 21% [F(2,21) = 5.683, *p* < 0.05], increased swimming behavior by 67% [F(2,21) = 4.270, *p* < 0.05] and had no effect on climbing [F(2,21) = 3.364, ns] (Fig. 3a).

Chronic treatment with HBK-15 (0.625 and 1.25 mg/kg) significantly decreased immobility of mice by 19% and 29% [F(3,28) = 6.929, *p* < 0.01], respectively (Fig. 3b). The compound administered for 21 days caused an increase in swimming behavior by 75% at the dose 0.625 mg/kg and by 111% at the dose 1.25 mg/kg [F(3,28) = 6.558, *p* < 0.01]. HBK-15 had no significant influence on climbing [F(3,28) = 0.356, ns].

Fluoxetine (10 mg/kg) administered for 21 consecutive days significantly decreased immobility by 27% [t(14) = 3.864, *p* < 0.01], increased swimming by 133% [t(14) = 4.604 ns], and had no influence on climbing behavior [t(14) = 0.483, ns] (Fig. 3c).

### The influence of studied compounds or fluoxetine on locomotor activity of mice after chronic treatment

Neither HBK-14 (1.25 and 2.5 mg/kg) [F(2,21) = 0.038, ns], HBK-15 (0.3, 0.625 and 1.25 mg/kg) [F(3,28) = 0.588, ns] nor fluoxetine (10 mg/kg) [t(14) = 0.436, ns] influenced locomotor activity in mice (Table 1).

**Table 1:** HBK-14, HBK-15 and fluoxetine did not influence locomotor activity of mice

Treatment	Dose (mg/kg)	Number of crossings ± S.E. M
Saline	-	397.6 ± 72.9
HBK-14	1.25	394.4 ± 45.2
	2.5	415.5 ± 53.6
Saline	-	424.5 ± 61.5
HBK-15	0.3	363.3 ± 65.4
	0.625	472.0 ± 47.8
	1.25	442.0 ± 63.7
Saline	-	430.6 ± 34.9
Fluoxetine	10	405.5 ± 45.9

HBK-14, HBK-15, fluoxetine or saline were administered intraperitoneally to mice for 21 consecutive days. The locomotor activity was recorded individually for each animal in activity cages between the 2nd and the 6th min (i.e., the time equal to the observation period in the forced swim test). Control groups received 0.9% NaCl (saline) Statistical analysis: one-way ANOVA (Newman-Keuls post hoc) when three or more groups were compared or Student’s t-test if two groups were compared; *n*=8 mice per group

### The influence of studied compounds on serotonin levels in murine hippocampi after acute and chronic treatment

**Fig. 4:**
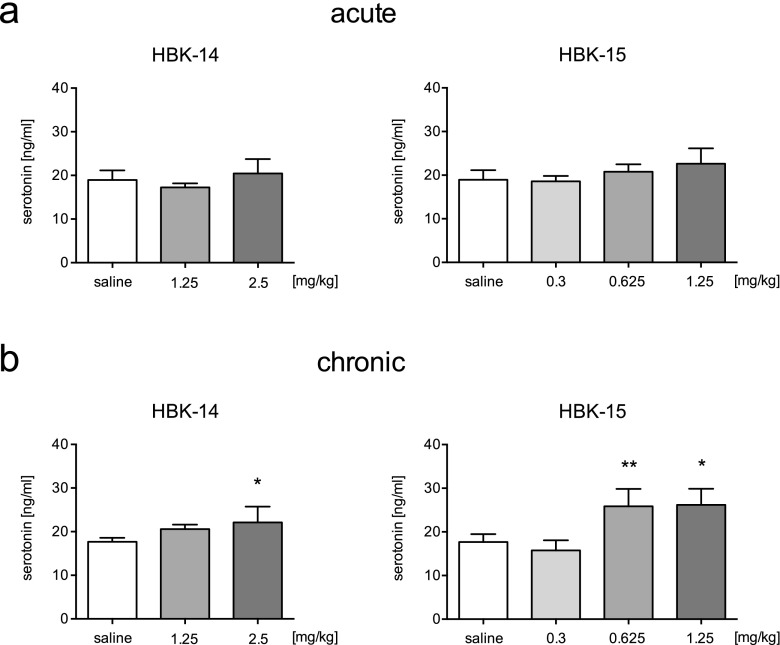
The influence on serotonin levels in murine hippocampi after acute (Panel a) or chronic (Panel b) treatment with HBK-14 or HBK-15. HBK-14, HBK-15 or saline were administered intraperitoneally to mice 30 min (Panel a) or for 21 consecutive days (Panel b) before tissue collection. Control groups received 0.9% NaCl (saline). Statistical analysis: one-way ANOVA (Newman-Keuls post hoc), *p < 0.05, **p < 0.01 vs respective saline-treated group; *n* = 6 mice per group

Acute treatment with the studied compounds showed no effect on the hippocampal serotonin levels (Fig. 4a). Administration of HBK-14 (2.5 mg/kg) and HBK-15 (0.625 and 1.25 mg/kg) for 21 days significantly increased serotonin levels in murine hippocampi by 25%, 46% and 48%, respectively (Fig. 4b).

### The influence on the guinea-pig ileum contraction induced by carbachol

**Fig. 5:**
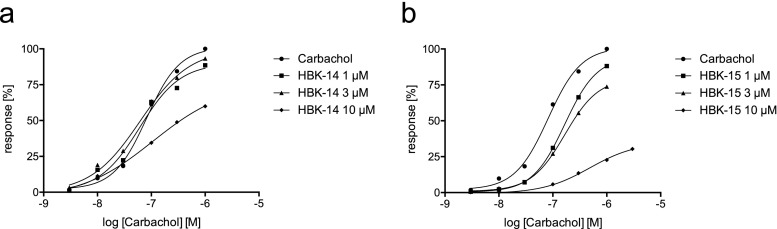
The effect of HBK-14 and HBK-15 on muscarinic receptor in isolated guinea-pig ileum. Concentration–response curves to carbachol in the absence or presence of increasing concentrations of HBK-14 or HBK-15. The results are expressed as the percentage of maximal response to carbachol in the corresponding concentration–response curve. Each point represents the mean ± S.E. M (*n* = 4–8)

Carbachol induced concentration-dependent guinea-pig ileum contractions; the pEC_50_ value (negative logarithm of the agonist concentration at which the response reached 50% of the maximal response) was 7.09 ± 0.05 (Fig. 5). Neither HBK-14 nor HBK-15 administered alone induced ileal contractions (data not shown). HBK-14 (1 and 3 μM) did not inhibit carbachol-induced contractions (Fig. 5a); however, at the concentration 10 μM it decreased the maximum effect of carbachol by 25%, which suggested a non-competitive antagonism. HBK-15 (1 μM) slightly shifted the carbachol response to the right without decreasing the maximal response, which indicated a competitive interaction with muscarinic receptors. The affinity expressed as the pK_B_ was 6.01 ± 0.01. Higher concentrations of HBK-15 (3 and 10 μM) decreased the maximum effect of carbachol by 21% and 65%, respectively (Fig. 5b). This suggested a non-competitive type of antagonism.

### The effect of studied compounds on learning and memory in the step-through passive avoidance task in mice

#### Evaluation of cognitive-enhancing properties in naïve mice

**Fig. 6:**
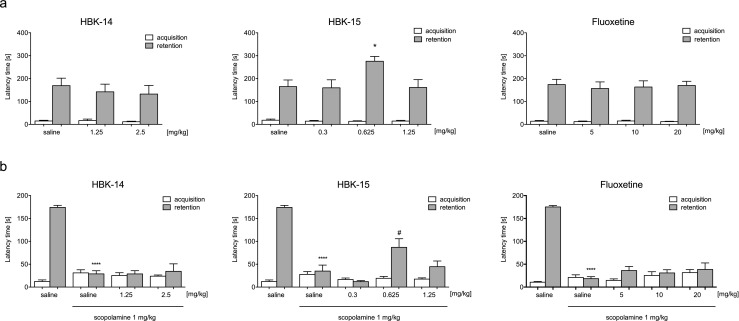
The effect of studied compounds or fluoxetine on learning and memory in the mouse step-through passive avoidance task. HBK-14, HBK-15, fluoxetine or saline and/or scopolamine were administered intraperitoneally to mice 30 min before the acquisition trial of the step-through passive avoidance test. The compounds were injected alone (Panel a) or jointly with scopolamine (Panel b). Control groups received 0.9% NaCl (saline). Statistical analysis: one-way ANOVA (Turkey post hoc), *p < 0.05, ****p < 0.0001 vs respective saline-treated group, #*p* < 0.05 vs respective saline + scopolamine-treated group *n* = 8–10 mice per group

In the acquisition trial of the passive avoidance task, none of the compounds affected latency times [HBK-14: F(2,27) = 0.393, ns; HBK-15: F(3,31) = 0.377, ns; fluoxetine: F(3,36) = 0.282, ns] (Fig. 6a). However, in retention trial HBK-15 (0.625 mg/kg) significantly increased latency time [F(3,31) = 3.564, *p* < 0.05] (Fig. 5a). In contrast, neither HBK-14 (1.25 and 2.5 mg/kg) nor fluoxetine (5, 10 and 20 mg/kg) influenced latencies in retention trial [F(2,27) = 0.305, ns and F(3,36) = 0.0.96, ns, respectively] (Fig. 6a).

#### The effect on scopolamine-induced cognitive dysfunction

In the acquisition trial, neither the studied compounds [HBK-14: F(3,36) = 2.446, ns, HBK-15: F(4,45) = 2.030, ns] nor fluoxetine [F(4,45) = 2.197, ns] affected latency times; however we observed significant differences in the retention trial (Fig. 6b). Scopolamine (1 mg/kg) compared with control (saline) significantly reduced latency times, and this effect was reversed by HBK-15 (0.625 mg/kg) [F(4,45) = 29.22, *p* < 0.0001] but not HBK-14 (1.25 and 2.25) [F(3,36) = 53.71, *p* < 0.0001] or fluoxetine (5, 10 and 20 mg/kg) [F(4,45) = 58.86, *p* < 0.0001](Fig. 6b).

## Discussion

In the present study we found that dual 5-HT_1A_ and 5-HT_7_ antagonists i.e. HBK-14 and HBK-15 possessed antidepressant-like activity and increased serotonin levels in the hippocampus after chronic treatment. HBK-15 possessed very weak cholinolytic properties, whereas HBK-14 did not block muscarinic receptors. Moreover, acute administration of HBK-15 enhanced memory of mice and significantly ameliorated memory impairment caused by scopolamine.

Although the data are inconsistent some studies show that 5-HT_1A_ and 5-HT_7_ receptor blockade might have potential in the treatment of major depressive disorder (reviewed in Pytka et al. 2016c). Researchers proved that 5-HT_1A_ receptor deficient mice were less immobile in the forced swim test than wild-type control (Parks et al. 1998; Heisler et al. 1998). This suggests that the lack of functional 5-HT_1A_ receptors favors a less depressed phenotype. Similarly, deletion of 5-HT_7_ gene (Guscott et al. 2005) as well as 5-HT_7_ receptor antagonists (Wesołowska et al. 2006) resulted in antidepressant-like effect in various animal tests. Altogether, these findings indicate that 5-HT_1A_ and 5-HT_7_ receptor antagonists may have potential as antidepressants.

Our previous studies revealed that dual 5-HT_1A_ and 5-HT_7_ receptor antagonists i.e. HBK-14 and HBK-15 possessed significant antidepressant-like properties after acute treatment (Waszkielewicz et al. 2015; Pytka et al. 2015a). Both compounds showed α_1_-adrenolytic properties, and only HBK-15 did not lower blood pressure at antidepressant-like doses after single administration (Pytka et al. 2016b). Interestingly, none of the compounds affected blood pressure after chronic administration (Pytka et al. 2016d). Here, we evaluated antidepressant-like activity of HBK-14 and HBK-15 after chronic administration in mice. Both compounds injected for 21 days showed antidepressant-like activities in the mouse forced swim test. None of them affected locomotor activity of mice; therefore, the observed effects were specific. Both compounds increased swimming, whereas had no influence on climbing behaviors. Since serotonin-targeting antidepressants increase swimming behavior in rodents (Nakatomi et al. 2008), we concluded that the serotonergic system was involved in the antidepressant-like effect of the studied compounds. HBK-15 showed stronger antidepressant-like properties than HBK-14, which is in agreement with our previous findings (Pytka et al. 2015a). Interestingly, HBK-15 administered chronically showed antidepressant-like properties at lower dose (0.625 mg/kg) than the dose active after acute treatment (1.25 mg/kg) (Pytka et al. 2015a). This is a common phenomenon for antidepressants, since antidepressant effect requires adaptive changes at the neuronal receptor level. Similarly, fluoxetine (Contreras et al. 2001) or *Hypericum perforatum* (Lozanondash and Rodriguez-L 2010) administered chronically showed antidepressant-like effects at lower doses than those active after acute injection.

According to the serotonin hypothesis a deficit in brain serotonergic activity might be a cause of depression or an important vulnerability factor in this disease. The findings are divergent, but some studies reported a decrease in serotonin or its metabolite levels in the brains of suicide victims or suicide attempters (for review, see Mann et al. 1989). The reduced levels of serotonin were most frequently found in brainstem. Although there are many other theories, the serotonin hypothesis is still to date, as most antidepressants in clinical use enhance the serotonergic neurotransmission.

Since many antidepressants elevate serotonin levels, we investigated the influence of studied compounds on the level of serotonin in the hippocampus after acute and chronic treatment. Scientists proved that hippocampus plays a central role in major depression (for review see: Campbell and Macqueen 2004). Our experiments showed that chronic (but not acute) treatment with the studied compounds caused a significant increase in the level of hippocampal serotonin. In both cases the increase in serotonin levels was in parallel with a decrease in the immobility of mice in the forced swim test. Since hippocampus plays crucial role in mood disorders and reduced serotonin levels might occur in depression, we believe that the fact that the studied compounds elevate hippocampal serotonin levels might be beneficial in depressed individuals.

Cognitive dysfunction is very common among patients with major depressive disorder and significantly affects their capacity to function (Darcet et al. 2016). Carvalho et al. (2015) suggested that several factors might contribute to cognitive dysfunction in major depressive disorder i.e. hyperactive hypothalamic-pituitary-adrenal axis, an increase in oxidative and nitrosative stress, increased apoptosis or diminished neurotrophic support. The cognitive impairments mostly occur during depressive episodes and include deficits in executive functions (attention, processing speed, cognitive flexibility) or learning and memory.

Bearing that in mind, in the second part of our studies, we examined the influence of dual 5-HT_1A_ and 5-HT_7_ antagonists on learning and memory in mice after acute administration. Since the blockade of muscarinic receptors may cause various unfavorable effects such as memory impairment, we first investigated potential cholinolityc properties of the studied compounds. Our experiments revealed that HBK-14 possessed no, and HBK-15 very weak and negligible cholinolytic activity. Both compounds reduced carbachol maxima at the concentration of 10 μM, which suggested a non-specific or additional site of interaction (most likely not related to muscarinic receptors). In comparison, previous experiments performed in our laboratory showed that pA_2_ value for atropine (cholinolytic drug) was 8.985 (Mogilski et al. 2015), which was a much higher value than pK_B_ (5.99) obtained for HBK-15.

The data on the effects of 5-HT_1A_ and 5-HT_7_ receptor ligands on learning and memory in rodents are ambiguous. Galeotti et al. (2000) as well as Tsuji et al. (2002) proved that stimulating 5-HT_1A_ receptor in mice promoted learning and memory. Opposite results presented Madjid et al. (2006), who reported that 5-HT_1A_ antagonists facilitated aversive learning in mice. Interestingly, the Authors also showed that 8-OH-DPAT (5-HT_1A_ agonist) displayed biphasic effect on retention times. Similarly, studies on the role of 5-HT_7_ receptor in cognitive function are also conflicting (reviewed in Meneses 2014). Nevertheless, 5-HT_7_^−/−^ mice showed impaired contextual hippocampal-dependent learning and decreased long-term synaptic plasticity in the hippocampus (Roberts et al. 2004). Moreover, both genetic and pharmacological inactivation of 5-HT_7_ receptor in mice resulted in deficits in hippocampus-associated spatial memory in the location recognition test (Sarkisyan and Hedlund 2009).

In the present study, we determined the influence of both dual 5HT_1A_ and 5-HT_7_ antagonists on learning and memory using the step-through passive avoidance test, which is a hippocampus-dependent memory task. In this test animals need to inhibit their natural tendency to enter the dark chamber. We showed that only HBK-15 administered alone possessed memory-enhancing properties in passive avoidance task. Moreover, the compound ameliorated memory deficits induced by scopolamine in this test. HBK-14 and fluoxetine were inactive in the step-through passive avoidance task. Interestingly, in both experiments HBK-15 displayed an inverted U-shaped dose-effect curve (0.625 mg/kg). This nonlinear relationship was frequently reported in pharmacological studies on cognitive functions and memory (Baldi and Bucherelli 2005). Although an inverted U-shaped dose-effect is widely described, it is very poorly understood. Scientists proposed several theories (e.g. arousal hypothesis), but the effect is most likely multifactorial and therefore difficult to explain.

Studies suggested that 5-HT_7_ receptor blockade resulted in the cognitive deficits in mice (Sarkisyan and Hedlund 2009; Freret et al. 2014). Eriksson et al. (2008) showed that even though SB-269970 (administered alone before the training session) had no effect on retention latency, it enhanced amnestic effects of 8-OH-DPAT in the passive avoidance test in mice. Since 8-OH-DPAT is also 5-HT_7_ receptor agonist, the Authors concluded that 5-HT_7_ receptor stimulation by 8-OH-DPAT counteracts 5-HT_1A_ receptor-mediated impairments in hippocampal-dependent contextual learning. Our findings are in agreement with the above studies, since our previous experiments revealed that HBK-15 compared with HBK-14 showed around three-fold stronger antagonistic properties at 5-HT_1A_ and weaker at 5-HT_7_ receptor (Pytka et al. 2015a). We believe that these slight differences in HBK-14 and HBK-15 receptor profiles might be responsible for the observed differences in the activity in passive avoidance task.

The limitation of our study was the fact that antidepressant-like activity of the compounds was not tested using animal model of depression (e.g. chronic unpredictable mild stress). Since depression is closely associated with stress, this would provide more insight into the therapeutic potential of studied compounds. Furthermore, we should also confirm memory-enhancing activity of HBK-15 using other tests, such as Morris water maze or Y-maze, as well as after chronic treatment.

Therefore, in future studies we plan to examine antidepressant-like properties of both compounds utilizing animal models of depression. We also intend to evaluate the influence of acute and repeated administration of HBK-15 on learning and memory using other behavioral paradigms in rodents.

## Conclusion

We demonstrated that both dual 5-HT_1A_ and 5-HT_7_ receptor antagonists (i.e. HBK-14 and HBK-15) possessed antidepressant-like activity and increased serotonin levels in the hippocampus after chronic treatment. None of the compounds displayed strong cholinolytic properties. Moreover, HBK-15 showed memory-enhancing activity and ameliorated memory impairment caused by scopolamine after acute administration. We think that dual 5-HT_1A_ and 5-HT_7_ antagonists might have potential in the treatment of depressive disorders with cognitive dysfunction, and therefore require extended studies to explore their pharmacological profile.
